# Preseptal and orbital cellulitis: how to identify and treat these conditions – and save lives

**Published:** 2024-02-09

**Authors:** Rilwan Muhammad, Fatima Kyari

**Affiliations:** 1 Consultant Ophthalmologist, University of Abuja Teaching Hospital, Gwagwalada, Abuja, Nigeria.

**Preseptal and orbital cellulitis can be difficult to distinguish clinically. It is important to know what to look for so you can identify which children are at risk of serious complications, as they may need support from a multidisciiplinary team.**

**Figure:**
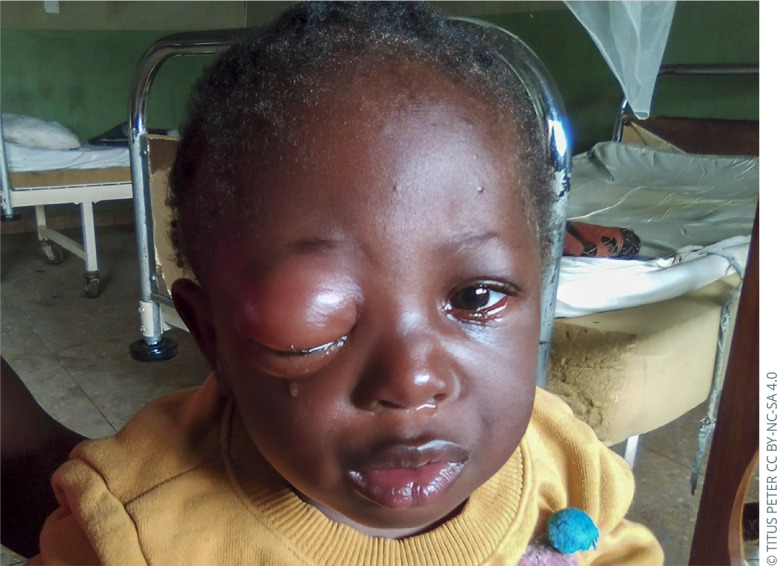
A child with orbital cellulitis who was admitted for treatment. nigeria

There are two main types of cellulitis that affect the tissues around the eye. Both are usually unilateral, affecting just one eye.

**Preseptal (sometimes known as periorbital)** **cellulitis** is an infection of the soft tissues around the eye **in front** of the orbital septum, the thin sheet of connective tissue which extends from the bony rim of the eye socket to the tarsal plates in the eyelids (Figure 1).^1^,^2^

**Orbital cellulitis** is an infection of the soft tissues **behind** the septum, inside the orbital cavity. Orbital cellulitis a serious and potentially life-threatening condition which requires an early diagnosis and prompt treatment with antibiotics, as the infection can spread into the brain.^1^,^2^

Some children can have features of both preseptal and orbital cellulitis.

## Causes of preseptal and orbital cellulitis

There is considerable overlap between the causes of preseptal and orbital cellulitis and the history alone cannot always distinguish the two. A computed tomography (CT) or magnetic resonance imaging (MRI) scan may be needed.^1^,^2^

**Figure 1:**
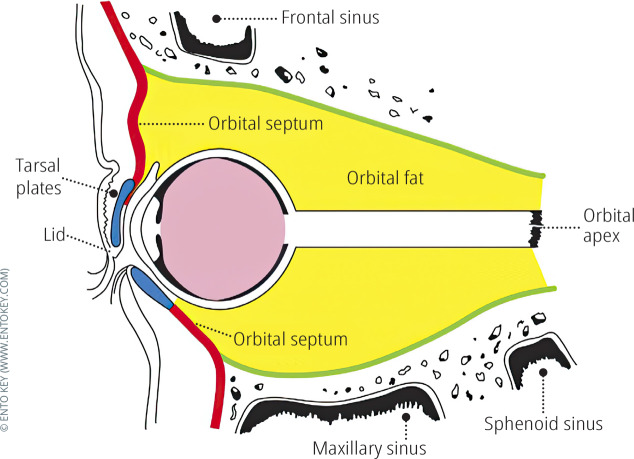
The septum (shown in red) is a sheet of thin connective tissue which extends from the bony prominence around the eye and attaches to the eyelids. It stops the orbital fat (the yellow area) around the back of the eye from coming forward into the eyelids.

**Figure 2:**
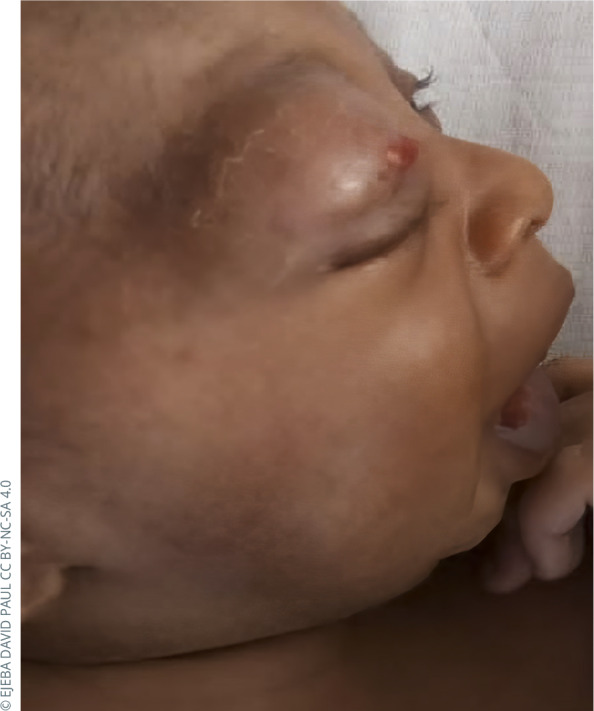
A neonate with severe preseptal cellulitis and lid abscess who was admitted and treated with intravenous antibiotics, with complete resolution. nigeria

**Preseptal cellulitis** can be caused by an insect bite, by an injury to the skin around the eye, or by spread of infection from a stye or an infected cyst in the eyelid (Figure 2). If there is no obvious eyelid cause, the infection may have spread from the sinuses – the bony spaces within the bones around the orbit (Figure 1).

**Orbital cellulitis** is more common and more severe in children and adolescents than in adults. In most cases (90%) the infection spreads into the orbit from the sinuses next to the nose (see Figure 1). Infection can also spread from eyelid skin, the lacrimal sac, or the teeth and gums, and may follow penetrating injuries of the orbit or surgery around the eye. Infection can also spread, via the bloodstream, from the upper respiratory tract and ears. In children, preseptal cellulitis can also, occasionally, progress to orbital cellulitis.

The most common bacteria responsible for orbital cellulitis are Gram-positive cocci: *Staphylococcus aureus*, *Streptococcus* species (*S. pyogenes* and *S. pneumonia*) and *Haemophilus influenzae*. Gram-negative bacilli may cause the infection in neonates, and following trauma.

## What to look for and ask about at the local or community level, and when to refer

Because the consequences of missing orbital cellulitis can be life-threatening, we recommend **urgent** referral of children where either orbital or preseptal cellulitis are suspected.

You should think of preseptal or orbital cellulitis, and not another cause of a swollen eyelid, if you see a child with any of the following:

- Red, swollen eyelids affecting either the right or the left eye
- Eye pain, or tenderness of one eye
- Drooping of the eyelid (ptosis)
- History of insect bite, lid trauma, or an upper respiratory tract, dental, or ear infection.

Ask the carer if the child has had an insect bite or trauma to the eyelid or whether they have, or have recently had, an upper respiratory tract infection, earache, or toothache.

If the eyelids are swollen in both eyes, and the eyes are watering and itchy, consider allergic conjunctivitis. In allergic conjunctivitis, the surrounding skin is usually a normal colour, whereas in an infective cause such as cellulitis, the surrounding skin is red.

If a newborn baby has these signs in both eyes, with thick discharge, they are likely to have conjunctivitis of the newborn due to chlamydia or gonococcal infection. Urgent referral is needed.

## Diagnosis and management in the eye department

The first thing to do is decide whether the child has preseptal or orbital cellulitis and not another condition causing marked lid swelling on one side. Other causes of swelling around the eye with proptosis in children include a retinoblastoma which has extended into the orbit, or a rhabdomysarcoma.

The next step is to decide whether the child has preseptal cellulitis or orbital cellulitis, as the management is different.

It can be difficult to decide whether a child has preseptal or orbital cellulitis. Table 1 summarises the different features. In orbital cellulitis, reduced vision and pupil abnormalities suggest that the optic nerve is involved, and that there is optic nerve compression.

**Table 1:** Features of preseptal and orbital cellulitis.^3^

**Feature**	**Preseptal cellulitis**	**Orbital cellulitis**
History	Insect bite or trauma around the eye	Upper respiratory tract infection, toothache, earache, headache
Protruding eye (proptosis)	Absent	Present
Eye movement	Normal	Painful, restricted
Visual acuity	Normal	Reduced in severe cases
Colour vision	Normal	Reduced in severe cases
Pupil abnormality (relative afferent pupil defect, RAPD)	Normal	Present in severe cases

An orbital CT or MRI scan may be needed to exclude non-infectious causes of the lid swelling and proptosis, such as retinoblastoma or rhabdomysarcoma, and to distinguish preseptal from orbital cellulitis. It is important to bear in mind that B scans cannot reliably differentiate between an orbital abscess and a tumour.

### Management of preseptal cellulitis

If the child has **preseptal celluliti**s, start treatment with oral broad-spectrum antibiotics such as flucloxacillin and co-amoxiclav. Follow local guidelines for soft tissue infection and adjust the dose according to the age and weight of the child. The child should be closely followed up.

### Management of orbital cellulitis

Children with orbital cellulitis should be admitted to hospital, as the management of orbital cellulitis requires a multidisciplinary approach, including paediatricians, ophthalmologists, and ENT surgeons.^1^,^2^,^4^ Investigations can include CT imaging of the brain and orbit, as well as full blood count, blood culture, and eye, nasal, and throat swabs.

Initial management may involve intravenous antibiotics such as flucloxacillin or ceftriaxone.

The child should be monitored closely in the hospital for signs of disease progression and the development of complications such as orbital and intracranial abscesses, meningitis, and cavernous sinus thrombosis.

The clinical signs and symptoms usually start to improve within 24 to 48 hours of intravenous antibiotics. Further investigations may be required if the clinical signs are not resolving, and/or to detect an underlying cause. These may include a CT scan of the brain, orbit, and paranasal sinuses to rule out subperiosteal and orbital abscesses and intracranial extension (sudural or brain abscesses and meningitis), and to monitor disease resolution or progression.

Further management might be required, such as surgical drainage of an orbital abscess with microbiological culture of fluid, particularly if there are signs of optic nerve involvement, such as reduced vision and abnormal pupil reflexes in the presence of proptosis).

Once the danger signs have resolved, the child can be discharged on oral antibiotics for 1–3 weeks. Explain to parents the importance of completing the course of antibiotics, what symptoms and signs to look out for, and what to do if that happens.

## Prevention & parent education

The risk of orbital cellulitis can be reduced by the prompt treatment of infections of the upper respiratory tract, sinuses, ears, mouth, and periocular skin.
